# *Haemophilus influenzae* and the lung (*Haemophilus* and the lung)

**DOI:** 10.1186/2001-1326-1-10

**Published:** 2012-06-14

**Authors:** Paul King

**Affiliations:** 1 Department of Respiratory and Sleep Medicine and Department of Medicine, Monash University, Monash Medical Centre, 246 Clayton Rd, Clayton, Melbourne, 3168, Australia

## Abstract

*Haemophilus influenzae* is present as a commensal organism in the nasopharynx of most healthy adults from where it can spread to cause both systemic and respiratory tract infection. This bacterium is divided into typeable forms (such as type b) or nontypeable forms based on the presence or absence of a tough polysaccharide capsule. Respiratory disease is predominantly caused by the nontypeable forms (NTHi). *Haemophilus influenzae* has evolved a number of strategies to evade the host defense including the ability to invade into local tissue. Pathogenic properties of this bacterium as well as defects in host defense may result in the spread of this bacterium from the upper airway to the bronchi of the lung. This can result in airway inflammation and colonization particularly in chronic obstructive pulmonary disease. Treatment of respiratory tract infection with *Haemophilus influenzae* is often only partially successful with ongoing infection and inflammation. Improvement in patient outcome will be dependent on a better understanding of the pathogenesis and host immune response to this bacterium.

## Review

*Haemophilus influenzae* was first identified by Pfeiffer in 1892, who (incorrectly) believed it was the cause of influenza [1]. It is an exclusively human pathogen and was the first bacterium to have its genome completely sequenced. This served as a precursor to the sequencing of the human genome.

*H*. *influenzae* is a component of the normal upper respiratory tract flora and is well recognized to be an important cause of systemic infection. It is also a major cause of a variety of respiratory conditions and has had a relatively low profile in this respect in comparison to some other pathogens; such as *Mycobacterium tuberculosisand Streptococcuspneumoniae*.

Recently there has been increasing recognition that this bacterium has a role in chronic lower respiratory tract inflammation. However the interaction between *H*. *influenzae* and the lung is still not well defined. A combination of bacterial pathogenic features and deficiency of host defense may permit this bacterium to establish infection in the lower respiratory tract resulting in inflammation and clinical disease. This review will discuss the role of *H*. *influenzae* in the lower respiratory tract in particular its role in bronchitis.

## Microbiology

**Figure 1:**
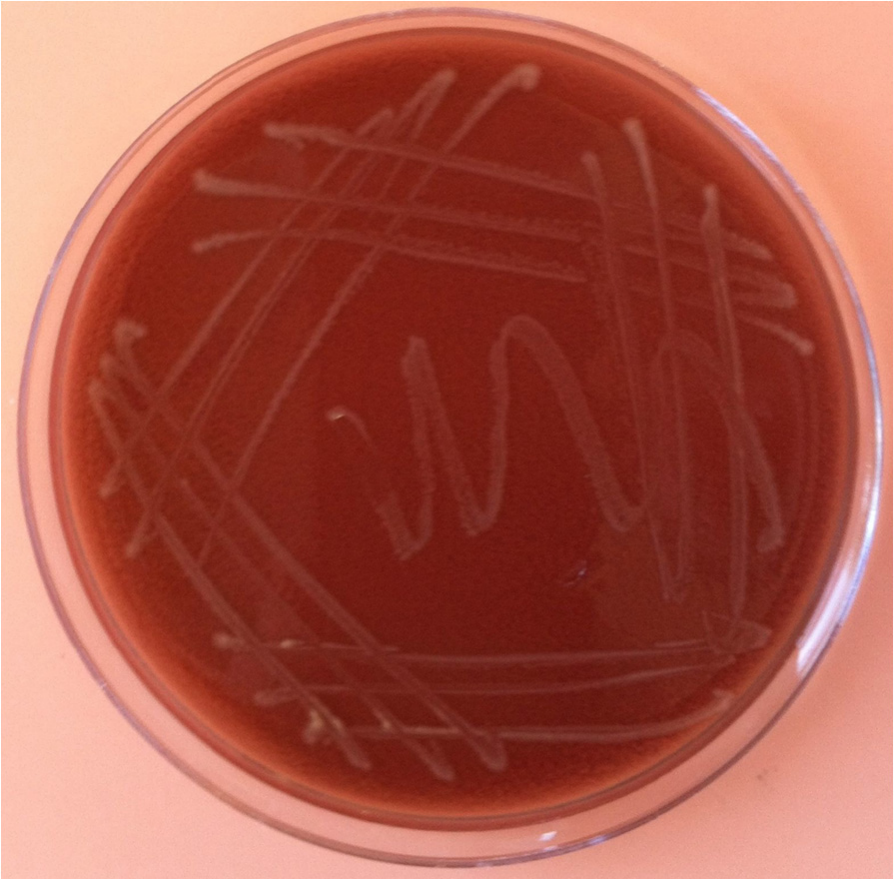
**Colonies of** ***Haemophilus influenzae*** **growing on a chocolate agar plate.**

*Haemophilus influenzae* is a gram-negative coccobacillus with a variable shape (pleomorphic). It grows both aerobically and anaerobically. Aerobic growth requires the presence of X (hemin) and V (nicotinamide adenine dinucleotide (NAD)) factors. In the laboratory it is classically grown on chocolate agar (Figure 1).

*Haemophilus influenzae* is divided into typeable and nontypeable strains on the presence or absence of a polysaccharide capsule. The typeable strains which have this capsule are classified into six serotypes (designated a to f) based on their ability to react with antisera against recognized polysaccharide capsules [2,3]. The type b form of *H*. *influenzae* (designated as Hib) is the most prominent typeable form and its capsule is composed of a linear ribosyl and ribotol phosphage polymer.

Nonencapulated forms are designated as nontypeable *Haemophilus influenzae* (NTHi). The NTHi strains are heterogeneous with a large number of different subtypes [4] that can be demonstrated on electrophoresis of outer membrane proteins or by enzymatic analysis. The NTHi strains are the main cause of *Haemophilus* respiratory infections. NTHi is classified into biotypes using indole, urease and ornithine decarboxylase.

## Epidemiology

Colonization of the upper respiratory tract with *H*. *influenzae* begins in infancy. Approximately 20 % of infants are colonized in the first year of life and this rises progressively over time [5]. By the age of 5–6 years more than 50 % of children will be colonized with this bacterium and most healthy adults (at least 75 %) will be [1]. This is a dynamic process with turnover of different strains. Children will have a strain for weeks to months, which will then be cleared and the acquisition of a new strain occurs [6]. Children will typically carry multiple strains simultaneously whilst adults will be colonized with only one strain [7].

This bacterium is spread by airborne droplets and contact with secretions [8]. Children in day centers have a particularly high incidence of infection and colonization with *H*. *influenzae*. The acquisition of new strains may be associated with acute exacerbations of airways disease [9]. The number of times a child is colonized with different isolates of NTHi is correlated with middle ear infection. The density of colonization is also a risk factor for the development of middle ear infection; this has been associated with lower levels of other commensal bacteria such as *Streptococcus viridans*[10].

The recent widespread use of the *H*. *influenzae* type B vaccine has caused a marked reduction in the prevalence of clinical disease arising from type b *H*. *influenza*e infection (from very common to rare). This has been associated with a proportionate decrease in the rate of nasopharyngeal colonization.

Most healthy adults will have upper airway colonization with *H*. *influenzae*. This process is dynamic and the predominant strains are nontypeable. The nasopharynx serves as a potential reservoir of infection from which this bacterium may spread to the lower respiratory tract.

## Pathogenesis

*Haemophilus influenzae* strains are divided into those with a capsule (e.g. the type b form) or the non-encapsulated strains (NTHi). The encapsulated strains primarily have a role in systemic infection in conditions such as meningitis. A principal defense against systemic *H*. *influenzae* infection is antibody-mediated complement killing. Normal serum is bactericidal for most strains of NTHi, but naturally-occurring bactericidal activity for encapsulated bacteria is much less common, particularly in children. In contrast NTHi strains rarely cause disease outside the respiratory tract and can be considered to be primary mucosal pathogens. The vast majority of respiratory disease arises from the NTHi strains. Important pathogenic mechanisms by which *H*. *influenzae* establishes respiratory tract infection will now be discussed in more detail.

### Mucociliary interactions

The mucociliary apparatus is a first-line structural defense against bacterial infection and H. *influenzae* strains have a variety of mechanisms which can influence its function. Outer membrane proteins such as P2 and P5 facilitate binding of the bacteria to mucus [11,12]. Lipooligosaccharide (a lipopolysaccharide that lacks the O-side chains and is abbreviated as LOS) is present in the cell wall of NTHi strains and has a significant effect on cilial function; Denny described that LOS produced inhibition of ciliary function and loss/death of ciliary mucosal cells [13]. Similar effects on ciliary function have been described from protein D which is a lipoprotein expressed on the surface of *H*. *influenza*[14].

### Attachment to respiratory mucosa

A key step in pathogenesis is the ability to adhere to the respiratory mucosa. NTHi appears to have a preference for nonciliated cells or damaged mucosa. There are several specific mechanisms which NTHi strains use to adhere to mucosa.

#### *Adhesins*

The adhesins are a common and important factor to facilitate epithelial attachment. These are present on a large proportion of NTHi strains and share some homology with adhesins expressed by *Bordatella pertussis*[15]. They are also a major target for serum antibodies to NTHi infection [16]. A number of investigators have demonstrated the importance of adhesins and there are 2 main subtypes HMW1 and HMW2 [17].

#### *Pili*

NTHi express pili which are rod-like projections on the surface which cause agglutination of red blood cells and attachment to respiratory tract epithelial cells [18]. There are 5 different types and these pili are present only on a small subset of strains of NTHi [19-21].

#### *Other factors*

Approximately 25 % of NTHi strains lack adhesins/pili but are still able to attach efficiently to respiratory epthilium. Two other factors that are important are the Hia and Hap proteins [22].

### Evasion of mucosal immunity

After attachment to the mucosal surface, strains of NTHi have a variety of different mechanisms which enhance persistence at the epithelial surface.

#### *Proteases*

Immunoglobulin (Ig) A is the main antibody subclass that prevents epithelial infection. IgA binds to bacteria and prevents mucosal attachment, inactivates toxins and facilitates cytotoxicity. The predominant IgA subclass is IgA1. NTHi secretes endopeptidases (Types 1 and 2), which cleave and neutralize IgA1 [23,24]. Nearly all NTHi strains express one of these IgA proteases which are highly effective in inhibiting IgA.

#### *Microcolony formation*

St Geme et al have demonstrated the ability of NTHi to form microcolonies on mucosal surfaces [8]. This property is likely to inhibit the function of secreted bacteriostatic products such as lactoferrrin and lysozymes and also potentially antibody function.

#### P*hase variation*/*antigenic drift*

Viruses such as influenza have well-documented abilities to frequently change cell structures and this is a key feature in their pathogenesis. *H*. *influenzae* is also able to lose or gain cell structures; a property called phase variation. Structures in which this occurs include LOS, adhesins and pili. This ability has significant implications for the function of antibodies and C-reactive protein [25].

In addition to phase variation, some strains of *H*. *influenzae* undergo antigenic drift which involves permanent change in amino acid sequences in some important immune structures/epitopes. This is best described in the context of the outer membrane protein P2 (which is strongly immunogenic) [26]. Other examples of antigenic drift involve P5 outer membrane protein and IgA1 proteases.

### Intracellular survival/invasion of local tissue

**Figure 2:**
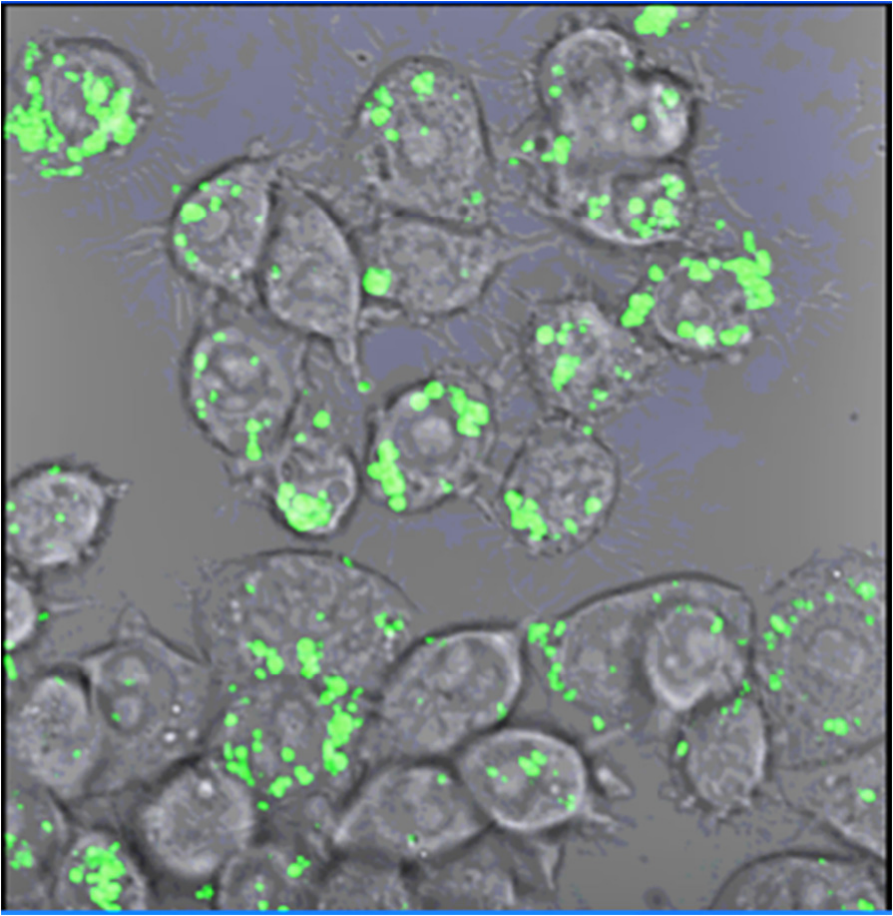
***H*.** ***influenzae*** **(stained with fluorescent dye) inside macrophages.**

A potentially very important pathogenic feature of *Haemophilus influenzae* is its ability to invade local tissue and survive intracellularly in the respiratory tract. This has been described in the context of nontypeable strains. The main cells targeted by NTHi appear to be macrophages and epithelial cells. There have been a number of studies which have demonstrated the in-vitro ability of NTHi to survive inside these cells for at least 72 hours [27-30]. An example of NTHi inside macrophages is shown in Figure 2.

Two early studies demonstrated the presence of NTHi between the epithelial cells of patients with chronic bronchitis and adenoidal inflammation [30,31]. Electron microscopy showed the disruption of intracellular junctions and the presence of bacteria between cells and in the phagocytic vacuoles of mononuclear cells. Forsgren et al demonstrated the presence of viable NTHi in mononuclear and epithelial cells obtained from children who had adenoid tissue resected as part of standard treatment [32].

Moller et al demonstrated extensive invasion of lung explants from patients with end-stage lung disease (including COPD and cystic fibrosis) with *H*. *influenza*; the organism was present in the epithelium, the submucosa of the bronchi, the bronchioles, the interstitium, and the alveolar epithelium in over half of the subjects [33]. Dromann et al have demonstrated the presence of *H*. *influenzae* in lung tissue in 40 % of subjects in all stages of COPD [34]. Another study found very high levels of intracellular NTHi present in subjects who had had exacerbations of COPD [35].

*Haemophilus influenzae* has a number of mechanisms that allow it to persist in the human host. The ability to be able to invade into lung tissue may be particularly important in the pathogenesis of this bacterium. Key mechanisms are summarized in Table 1.

**Table 1:** **Important pathogenic features of nontypeable** ***Haemophilus influenzae***

Mucociliary interactions	Binding to mucus
	Inhibition of ciliary function/death of ciliary cells
Attachment to respiratory mucosa	Adhesins
	Pili
	Hia/Hap proteins
Evasion of mucosal immunity	IgA proteases
	Microcolony formation
	Phase variation/antigenic drift
Intracellular survival/invasion of local tissue	Survival inside mononuclear phagocytes and epithelial cells
	Extensive invasion of lung parenchyma

## Immune response

NTHi is present in the nasopharynx of most healthy adults but only causes clinical disease in a minority of subjects it infects. Therefore the nature of the respiratory tract immune/inflammatory response may be very important in the pathogenesis of this bacterium. NTHi causes strong stimulation of both innate and adaptive immunity.

### Innate immune responses

Innate immunity serves as the first-line of defense against infection and is comprised of both structural and cellular defenses.

Structural defenses have a number of components including cough, barrier function and the mucociliary apparatus. Impairment of mucocilary function as occurs in cystic fibrosis and immotile cilia syndrome, is associated with lung *H*. *influenzae* infection.

The cellular innate immune response is a rapidly evolving area. It is mediated by neutrophils and particularly macrophages, which recognize bacterial pathogens by toll-like receptors (TLRs). The outer membrane proteins of *H*. *influenzae* such as P2 and P6 strongly activate innate immunity. P6 activates macrophages to produce interleukin (IL) 8 and tumor necrosis factor alpha (TNF-α) [36,37]. P6 is also important for the migration of dendritic cells. NTHi lysate (i.e. killed/inactivated bacteria) upregulates the production of nuclear transcription factor-kappa β (NF-κβ), which is a key driver of inflammation [38]. Epidermal growth factor receptor (EGFR) has also recently been shown to be important in NF-κβ activation [39].

The NF-κβ pathway is principally activated through toll-like receptors (TLRs) which function as pattern receptors (e.g. for bacterial motifs). Activation of TLRs by bacterial motifs drives an inflammatory response, which is designed to clear infection. Both TLR-2 and TLR-4 have been shown to be important in immune responses to NTHi [40,41].

TLR-4 primarily recognizes lipopolysaccharide (LPS) a component of the gram-negative cell wall. NTHi has lipooligosaccharide (LOS), which is very similar to LPS but lacks O-antigen units. Activation of TLR4 results in the production of a variety of inflammatory mediators by the macrophage, which have a key role in initiating innate immunity.

TLR-2 is also expressed on the surface of innate and immune cells. Outer membrane P6 activates TLR-2 with up-regulation of NF-κβ and the production of inflammatory mediators.

### Adaptive immune responses

The adaptive immune response develops after the innate response and is particularly important in chronic infectious disease. It is primarily mediated by B lymphocytes (humoral immunity) and T lymphocytes (cellular immunity).

Studies have demonstrated that the great majority of healthy subjects and those with chronic airways disease have strong antibody responses to NTHi. It has also been shown that complement is bactericidal for NTHi. Antibody causes activation of the terminal attack complex of complement and this is very effective in killing NTHi [42]. This mechanism may explain why NTHi is primarily a mucosal pathogen that rarely spreads beyond the respiratory tract in contrast to the typeable forms such as Hib which are protected by the tough polysaccharide capsule and frequently cause systemic disease. Hypogammaglobulinaemia has been shown to be an important risk factor for systemic infection with NTHi.

T cells have a key role in the protection against intracellular infection. Both T helper (Th) cells and cytotoxic T (CTL) cells secrete cytokines (which drive inflammation) and produce cytotoxic mediators. The ability of T cells to proliferate to NTHi stimulation influences clinical disease. Patients with obstructive airways disease have deficient T-cell responses to NTHi when compared to healthy controls specifically; 1), decreased Th1 cell function, particularly production of interferon gamma (IFN-γ) & CD40 ligand (CD40L) [43] and 2), decreased CTL cell function, particularly production of IFN-γ [44]. In addition macrophage killing of NTHi is enhanced by the addition of IFN-γ & CD40L [45]. A recent study has confirmed that Th1 cell responses to NTHi in COPD are deficient [46] and TLRs appear to be important in this mechanism.

There are two other important situations, which have potentially significant effects on the immune response to NTHi in the lung. They are cigarette smoke and viral infections. These will now be discussed in more detail.

### Effect of cigarette smoking on the lung immune response

Cigarette smoking is the key risk factor for the development of COPD and has a significant effect on the lung immune response [47]. Smoking directly damages the respiratory epithelium and inhibits mucociliary function [48,49]. It has a wide variety of effects on alveolar macrophages, dendritic cells and lymphocytes which inhibit the ability of the lung to clear infection [47]. Smoking has been found to be associated with increased lung inflammation following challenge with NTHi [50].

### Interaction between viral infection and *Haemophilus influenzae*

In clinical practice there is a frequent association between viral infection and bacterial infection. In the context of *H*. *influenzae* infection the two best-described associations have been with 1), epidemic influenza and 2), rhinovirus.

Exacerbations of COPD are frequently associated with combined viral and bacterial infection and the most common co-infection is the combination of rhinovirus and *H*. *influenzae*, which is also associated with increased severity of exacerbations [51,52]. There is preliminary evidence from laboratory studies that rhinovirus may interact with NTHi. Rhinovirus suppresses host macrophage interleukin 1 responses to NTHi [53] and enhances migration of NTHi across epithelial cells [54].

A major cause of death in epidemic viral influenza is secondary bacterial lung infection. Outbreaks are associated with different bacterial infections. The “Spanish flu” outbreak following World War 1 was associated with a high incidence of *H*. *influenzae* infection [55,56]. Viral influenza causes significant damage to the respiratory epithelial barrier and this may be an important mechanism of secondary bacterial infection.

## Clinical features

Infection of the lower respiratory is predominantly by the nontypeable strains of *Haemophilus influenzae* (NTHi). The dominant manifestation is bronchitis, which may be acute or chronic and has been well described in the context of COPD, bronchiectasis and cystic fibrosis. Exacerbations are an important feature of these forms of bronchitis and are characterized by episodes of acute clinical deterioration usually in association with increased airway inflammation. Additionally this bacterium is also a major cause of pneumonia.

### Chronic obstructive pulmonary disease

COPD is a major health problem and the fourth leading cause of mortality worldwide. It is characterized by the presence of airway inflammation that is most pronounced in the bronchial wall. This bronchial wall inflammation persists despite the cessation of smoking.

The most common cause of chronic bacterial airway colonization in patients with COPD is NTHi accounting for up to half of all isolates [35,57-60]. The major bacterial cause of exacerbations of COPD is NTHi, 25 to over 80 % of exacerbations are associated with *H*. *influenzae*[35,57-59]. In addition the bacterial load of NTHi in lung airways has been shown to contribute to airway inflammation in stable chronic bronchitis [61]. Colonization with NTHi is associated with more severe COPD exacerbations [62].

Exacerbations of COPD from NTHi can arise from the acquisition of new strains [9](presumably from the upper airway) or from existing colonizing strains.

The exact role of NTHi in the inflammatory process is still not well defined but it is possible that NTHi may invade into the bronchial wall to activate T cells and contribute to inflammation. A murine model of COPD has recently demonstrated that the addition of an NTHi lysate induces the formation of lymphoid follicles in the bronchi [63].

### Bronchiectasis and cystic fibrosis

Bronchiectasis is characterized by persistent bacterial infection that causes lung damage and also results in chronic airflow obstruction [64]. The bacterial infection generally appears to result as a consequence of a defect in host defense which results in chronic bacterial airway infection. The dominant bacterium in bronchiectasis is NTHi and this is a major driver of lung inflammation. There is considerable overlap between bronchiectasis and COPD.

The principal manifestation of cystic fibrosis (CF) is severe and progressive bronchiectasis, which results in respiratory failure. It is thought that a defect in mucociliary function (through the cystic fibrosis transmembrane conductance regulator (CFTR) gene) results in progressive airway infection. NTHi is prominent in the early stages of CF; in later stages other bacterium such as *Pseudomonas* are predominant. However Moller et al described *H*. *influenzae* to be the dominant bacterium in the lung parenchyma of explants from CF patients. Therefore in the later stages of CF NTHi may still be important in parenchymal lung infection.

### Pneumonia

NTHi is also one of the leading causes of pneumonia in adults (with *Streptococcus pneumoniaeand Moxarellacatarrhalis*). Most patients with NTHi pneumonia have bronchopneumonia with cough, fever and systemic upset. The illness is generally less fulminant than that with *S*. *pneumoniae* and commonly occurs in patients with underlying lung disease (particularly COPD).

**Figure 3:**
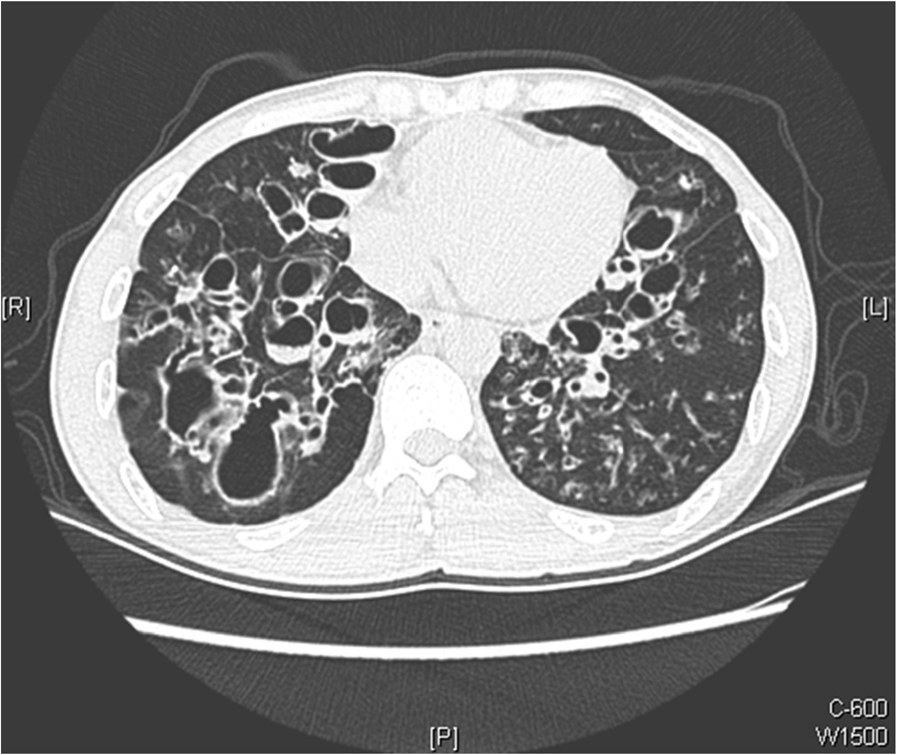
**Computed tomography (CT) scan of a subject with severe lower lobe bronchiectasis and recurrent isolation of** ***H*.** ***influenzae*** **from sputum.**

**Figure 4:**
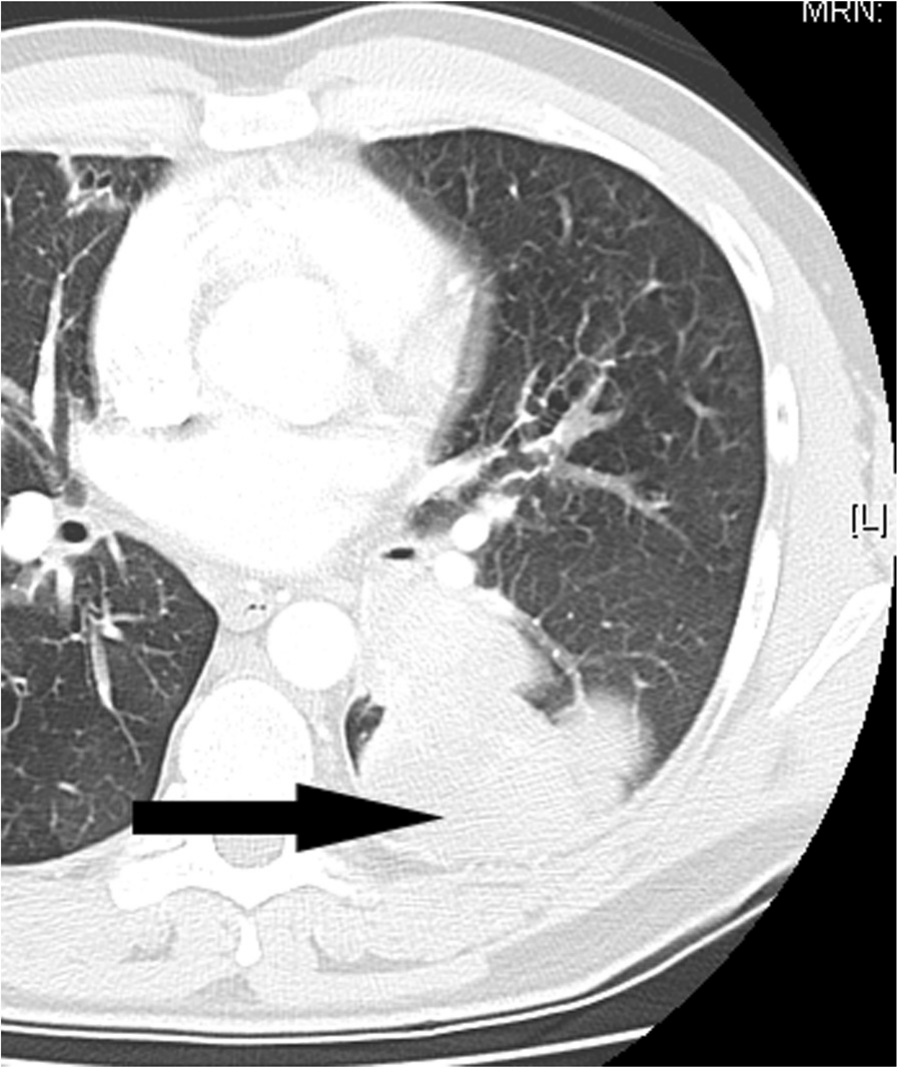
**Computed tomography scan taken of a patient with a lung abscess that required surgical removal.***H*. *influenzae* was isolated from the lung tissue.

Two examples of patients with *H*. *influenzae* infection are shown in Figures 3 and 4.

### Possible role of NTHi in lung cancer

Smoking is the primary risk factor for both chronic obstructive pulmonary disease (COPD) and lung cancer. Only a minority of smokers develop COPD and the presence of COPD has been demonstrated to be an important risk factor (independent of the risk of smoking) in the development of lung cancer. It has been speculated that NTHi-driven inflammation in the context of COPD may be a risk factor for the development of lung cancer [65].

There have been several mechanisms described which may suggest a role for NTHi-driven inflammation as a risk factor for lung cancer. NTHi activates proliferative and antiapoptotic pathways [38,66] and promotes lung cancer in a Clara cell-targeted mouse model of lung cancer [67]. Interleukin-6 and Il-17 generated in response to NTHi may be important risk factors as well.

## Treatment

Treatment of *Haemophilus influenzae* infection should be straightforward as this bacterium is usually sensitive to most standard antibiotics. In practice though treatment is often only partially successful with recurrent/non-clearing infection. Particularly in the context of chronic bronchitis (e.g. as in COPD and bronchiectasis) NTHi often appears to have established a niche in the airway, which makes it relatively resistant to chemotherapy.

### Use of antibiotics

Oral antibiotics such as β-lactams are appropriate first-line therapy for most patients. A proportion of *H*. *influenzae* isolates produce β-lactamase (this varies markedly between different locations) and in this circumstance, extended spectrum cephalosporins, amoxicillin-clavulinic acid, trimethoprim-sulfamethoxazole, tetracyclines, quinolones and macrolide antibiotics are appropriate therapeutic choices. In Spain and Japan, β-lactamase negative, ampicillin-resistant (BLNAR) strains are prevalent. These BLNAR strains are associated with a high incidence of pneumonia [68,69]. For hospitalized patients particularly if there is associated respiratory failure the parenteral route of administration may be preferred.

For patients who have repeated isolation of *H*. *influenzae* despite the use of appropriate antibiotics consideration should be given to the use of longer-term antibiotics or those with good intracellular penetration. As discussed previously NTHi has the ability to live intracellularly and thus be protected from a number antibiotics particularly the β-lactams [70]. Agents with good intracellular penetration include macrolides, tetracyclines and quinolones. In the author’s experience, longer courses of antibiotics with good intracellular penetration may be helpful (although this has potential issues of resistance).

### Vaccination

The vaccine to type B encapsulated *H*. *influenzae* induces humoral immunity to the capsule and has been highly effective in reducing the incidence of disease from this pathogen. This vaccine is now standard for children in industrialized countries. Vaccination is usually given at 2 months, 6 months and 12–15 months.

In contrast to Hib vaccination for NTHi strains is not well established. NTHi infection occurs from a wide variety of different and very heterogeneous strains and the protective immune response has not been clearly defined. There have been a number of published studies measuring the effect of vaccination in animal models. A major problem with the use of animal models is that NTHi is an exclusively human pathogen, which generally does infect animals well.

An oral vaccine consisting of multiple strains of killed NTHi has been used with some reduction in exacerbations [71]. More recently 2 trials of a new form of oral NTHi vaccine have demonstrated a reduction in exacerbations [72,73]. Subjects were given 3 doses of inactivated NTHi on a monthly basis with changes in the expression of T cell responses and specific immunoglobulin production.

## Conclusions

**Figure 5:**
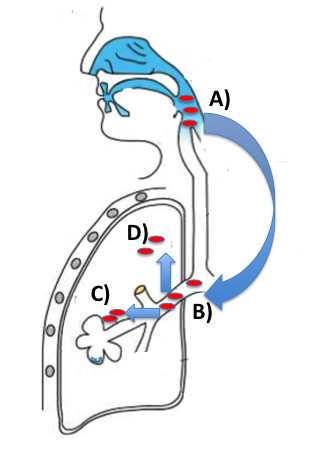
**The potential spread of nontypeable strains of*H*.*influenzae*(NTHi) into the lung.A**), Most healthy subjects have nasopharyngeal colonization with NTHi. A combination of bacterial pathogenic mechanisms and defects in host defense may allow NTHi to move down into the lower respiratory tract **B**). In the bronchi, NTHi may cause acute exacerbations of airway disease and/or chronic colonization. **C**), The bacterium may invade into bronchial cells such as macrophages or epithelial cells or **D**), may in more advanced lung disease invade into the parenchyma.

*H*. *influenzae* is present in the nasopharynx of most healthy adults. Defects in host defense and pathogenic mechanisms of the bacteria may result in migration into the lower respiratory tract. This results in bronchitis, which may be acute or chronic. Invasion into respiratory tract cells, the bronchial wall and parenchyma of the lung may then occur. This process is summarized in Figure 5.

This bacterium is increasingly being recognized as a major cause of chronic infection/inflammation in the context of COPD. In the author’s opinion the establishment of *H*. *influenzae* infection in the airway may lead to a self-perpetuating inflammatory process that has a major role in the development of airway obstruction. Better understanding of the pathogenesis of *Haemophilus influenzae* in the respiratory tract is likely to lead to more effective treatment; in particular the use of vaccination.

## Competing interests

The author declares that he has no competing interests.
